# FDA and EMA Approvals of New Breast Cancer Drugs—A Comparative Regulatory Analysis

**DOI:** 10.3390/cancers12020437

**Published:** 2020-02-13

**Authors:** Chandra P. Leo, Bettina Hentschel, Thomas D. Szucs, Cornelia Leo

**Affiliations:** 1 HBM Partners, 6300 Zug, Switzerland; 2 Institute for Medical Informatics, Statistics and Epidemiology (IMISE), University Leipzig, 04107 Leipzig, Germany; bettina.hentschel@imise.uni-leipzig.de; 3 European Center for Pharmaceutical Medicine, University of Basel, 4056 Basel, Switzerland; thomas.szucs@unibas.ch; 4 Interdisciplinary Breast Center, Kantonsspital Baden, 5404 Baden, Switzerland; cornelia.leo@ksb.ch

**Keywords:** breast cancer, drug approvals, drug devFelopment, EMA, European Medicines Agency, FDA, Food and Drug Administration, oncology, regulatory affairs

## Abstract

Breast cancer is the most common cancer in women worldwide and the solid tumor type for which the highest number of drugs have been approved to date. This study examines new drug approvals for breast cancer by the United States Food and Drug Administration (FDA) and the European Medicines Agency (EMA), based on an analysis of regulatory documents from both agencies for the period from 1995 to 2018. Of the 29 breast cancer drugs approved over this time span, 17 received positive decisions from both the FDA and EMA, including all drugs licensed after 2008. Nineteen of the 25 FDA-approved drugs, but none of the EMA approvals, benefited from special regulatory pathways (such as fast track, breakthrough therapy, or priority review). In the U.S.A., four accelerated approvals were granted (of which one, for bevacizumab, was later revoked), while only two drugs received provisional approvals following EMA review. New breast cancer drugs were approved approximately twelve months earlier in the United States than in Europe. These results suggest that a broader use of special regulatory pathways by EMA could help to accelerate access to novel drugs for European breast cancer patients.

## 1. Introduction

Globally, breast cancer is the most common malignancy diagnosed in women, accounting for a quarter of all new cancer cases in the female population [1]. Worldwide, over 1.5 million new cases are recorded each year, with more than 500,000 estimated deaths due to breast cancer. As a consequence, the development and approval of new drugs for the treatment of this condition continue to be important priorities for patients, clinicians, regulators, and the pharmaceutical industry [2].

In a previous study, we analyzed all United States Food and Drug Administration (FDA) approvals of new breast cancer drugs between 1949 and 2018 [3]. We showed that over this 70-year period, breast cancer accounted for the highest number of FDA drug approvals among all solid tumor entities. Furthermore, we found that the rate of new drug approvals for breast cancer has dramatically increased over the last 30 years, compared to earlier decades, in part driven by the development of targeted therapeutics [3]. This trend matches the acceleration of new drug approvals seen across the field of oncology as a whole [4,5], which results from the impressive progress in translational cancer research and from the evolving regulatory environment [6,7]. Together, these factors have profoundly changed the way in which new cancer drugs are discovered, studied, reviewed, and approved [8,9,10].

Since the review and approval of new drugs lie in the responsibility of national or regional authorities, approval decisions and timelines may diverge between different geographies. Previous studies comparing drug approval processes and outcomes in the United States and other countries/regions demonstrated that the U.S.A. approval process is generally the fastest [11,12,13]. Only very few publications specifically address the approval of new oncology drugs across different regulatory regions, and these are restricted to certain drug classes and/or date back to periods with differing regulatory environments for cancer drugs [14,15]. To our knowledge, no focused and comprehensive analysis of new approvals for breast cancer drugs in the U.S.A. and Europe has been conducted to date.

Therefore, the aim of this study was to compare U.S.A. and European approvals of new drugs for breast cancer during the time period from 1995 to 2018, based on a review of FDA and European Medicines Agency (EMA) regulatory documents. In particular, we examined the concordance/discordance of approval decisions, the use of special regulatory programs, the time interval between approval dates, and the duration of the respective review processes.

## 2. Results

In 1995, the European Medicines Agency (EMA) was established in order to harmonize the approval of medicinal products in the European Union. This gave EMA the authority to evaluate novel cancer drugs through a centralized procedure, thus becoming the European counterpart to the United States FDA. Table 1 summarizes all initial approvals of novel drugs for use in breast cancer during the time period from 1995 to 2018.

### 2.1. Concordance/Discordance of Approval Decisions

Of the 29 drugs we identified, 17 received an approval for breast cancer in both jurisdictions, following review by the FDA and EMA, respectively. Six additional drugs had, by the time of their FDA approval, already been approved in (parts of) Europe through the non-centralized regulatory pathways used before the establishment of the EMA. In two cases, drugs received FDA approval, but no European approval: in 2007, the FDA included the “reduction in risk of invasive breast cancer” in high-risk postmenopausal women in its label for the SERM (selective estrogen receptor modulator) raloxifene, while the corresponding EMA label was restricted to the treatment and prevention of osteoporosis. Furthermore, in 2007, the microtubule-stabilizing agent ixabepilone became one of the last cytostatic/cytotoxic drugs to receive FDA approval for breast cancer, but EMA’s CHMP (Committee for Medicinal Products for Human Use) came to a negative assessment of the benefit–risk balance and the European MAA (marketing authorization application) was subsequently withdrawn.

Conversely, three breast cancer drugs that were centrally approved in Europe did not receive an equivalent FDA approval: two of them were liposomal formulations of the topoisomerase inhibitor doxorubicin. While the non-PEGylated liposomal doxorubicin was never approved in the United States at all, the PEGylated version only received FDA approval for malignancies other than breast cancer. The third of these drugs was ibandronate, a bisphosphonate with an osteoporosis indication. In Europe, but not the United States, ibandronate received an additional label for the “prevention of skeletal events in patients with breast cancer and bone metastases”. Among the 29 drugs in Table 1, 21 (72.4%) received their first-ever approval in breast cancer. Only eight drugs had previously been approved in other indications, namely goserelin, gemcitabine, raloxifene, ibandronic acid, doxorubicin (PEGylated liposomal), bevacizumab, everolimus, and olaparib.

### 2.2. Use of Special Regulatory Programs

Over the last four decades, FDA and EMA have established a range of special designations and pathways to expedite the development and approval of promising new drugs addressing unmet needs and/or offering major advances in treatment.

In the United States, 19 of the 25 new approvals receiving a breast cancer label between 1995 and 2018 benefited from one or more special designations, namely fast track (six drugs), breakthrough therapy (three), priority review (15), and orphan drug (four). Notable examples include the HER2-targeting agents trastuzumab and ado-trastuzumab emtansine (both granted fast track and priority review) and the CDK4/6 inhibitors palbociclib, ribociclib, and abemaciclib (each received breakthrough therapy and priority review designations). In contrast, none of the 21 drugs achieving a centralized EU marketing authorization for breast cancer did so under PRIME, accelerated assessment, or orphan drug designation (see Table 1).

Moreover, both the FDA and EMA possess special pathways allowing for the provisional approval of drugs based on surrogate endpoints and/or less comprehensive data than normally required. In the United States, three drugs receiving their first breast cancer label were granted such an accelerated approval which was converted to a standard approval following the review of additional data: docetaxel (converted after 21.6 months), capecitabine (converted after 6.0 months), and palbociclib (converted after 5.7 months). In Europe, docetaxel received a marketing authorization “under exceptional circumstances” (converted to standard approval after 31.3 months), whereas lapatinib was given a conditional marketing authorization (converted after 80.3 months).

In total, among the 17 drugs with dual approvals, 14 (82.4%; 95% CI: 56.6–96.2%) received one or more special designations/pathways from the FDA, compared to only two (11.8%; 95% CI: 1.5–36.4%) from EMA, a difference that was highly statistically significant (*p* < 0.0001). Specifically, the programs drastically reducing review times were also more frequently applied in the U.S.A., with 12 cases of priority review (70.6%; 95% CI: 44.0–89.7%) compared to Europe, which had no cases of the equivalent accelerated assessment (0%; 95% CI: 0–19.5%), again a highly statistically significant difference (*p* < 0.0001).

In only one case was a provisional approval for a breast cancer drug subsequently withdrawn: after initial approvals in colorectal and non-small cell lung cancer, the FDA had granted the VEGF inhibitor bevacizumab accelerated approval for breast cancer in February 2008, while demanding additional data “to further define the degree of clinical benefit”. Following the review of these study results, the FDA withdrew the breast cancer indication in November 2011. In contrast, the European Commission had already in March 2007 granted bevacizumab a full approval for use in breast cancer, which still remains in place today.

### 2.3. Relative Timing of Approval Decisions and Duration of Review Processes

**Figure 1:**
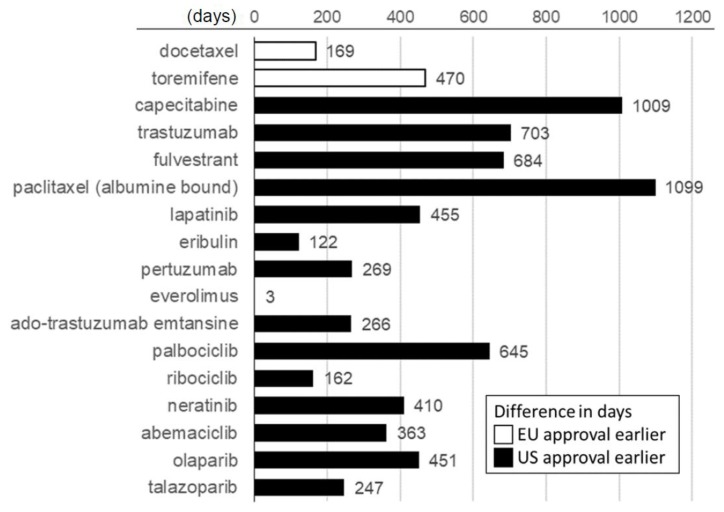
“Approval lag” between the U.S.A. and Europe, based on the relative timing of first approval for breast cancer.

Our analysis of the timelines to approval also revealed widespread differences between the two jurisdictions. As shown in Figure 1, among the drugs (excluding bevacizumab) that maintained dual approval, a positive FDA decision was issued before the European one in 15 of the 17 cases. The two exceptions were docetaxel in 1995 and toremifene in 1996, the first two breast cancer drugs to be approved by the newly established EMA. In total, we observed a median difference of 363 days (95% CI: 162–645 days; *p* = 0.003) in favor of an earlier U.S.A. approval date.

**Figure 2:**
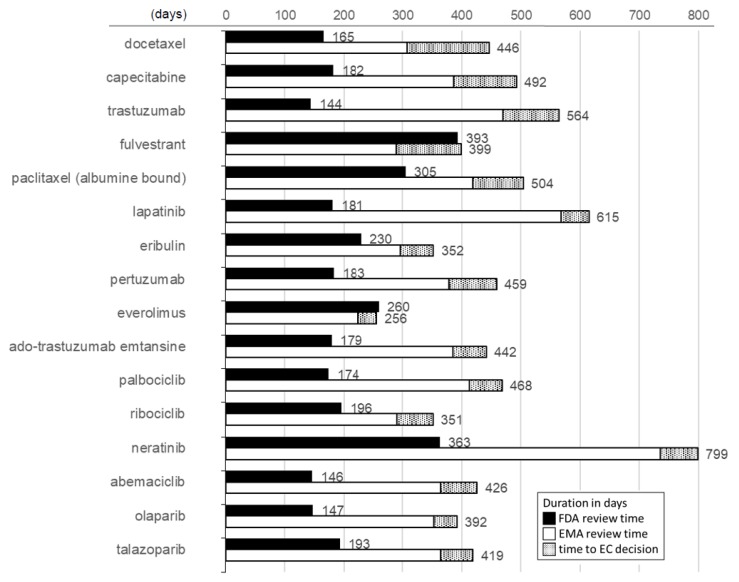
Duration of approval process in the U.S.A. vs. Europe, based on time from NDA (New Drug Application) or MAA (Marketing Authorization Application) submission to approval.

In order to analyze the factors behind these differing approval dates, we compared the time span between dossier submission and positive approval decision, which in the case of EMA includes an additional time interval until a CHMP recommendation is confirmed by a subsequent European Commission (EC) decision (Figure 2). For 15 of the 16 analyzable drugs with dual approvals (toremifene excluded, see Methods), the FDA review time was shorter than the European process, while in the case of everolimus, the difference was only four days in favor of the EU.

Altogether, FDA approval processes were shorter than the EU ones by a median of 269.5 days (95% CI: 190.5–295.5 days; *p* < 0.0001). In Europe, the time span just from MAA submission to positive CHMP opinion (i.e., net of the additional time to an EC decision) had a median duration of 371 days (range: 224–735 days), which was still significantly longer than the total FDA review time by 199.5 days (95% CI: 114–218 days; *p* < 0.001). In Europe, the additional time from positive CHMP opinion to final EC decision had a median duration of 61.5 days (range: 32–138 days). With a median of 182.5 days (range: 144–393 days), the FDA’s median interval from NDA (new drug application) submission to positive approval decision was less than half as long as the median EMA review process net of the time to EC decision.

## 3. Discussion

As shown in our earlier study, breast cancer was the solid tumor entity with the highest number of newly FDA-approved drugs during the 70-year period from 1949 (the year of the first-ever approval of an oncology drug, mechlorethamine) to 2018 [3]. If one includes the field of hematologic oncology, breast cancer resides in third place, behind leukemias and lymphomas. More than two thirds of the drugs analyzed in our study received their first-ever approval for breast cancer and more than half (16 of 29) have to date not been approved for additional indications. This high degree of regulatory activity reflects the medical and commercial importance of breast cancer as the most frequently diagnosed malignancy in women. 

In our present analysis, we did not include European approvals of breast cancer drugs during the pre-EMA era. These had taken place under a different set of non-centralized structures and procedures and were therefore not directly comparable to the current EMA, nor to the FDA system. With the establishment of the EMA in 1995, Europe gained a central approval process for new drugs that is broadly comparable to that of the United States’ FDA [16]. Notwithstanding efforts to harmonize regulatory processes on an international level, EMA’s structures and procedures diverge in various important aspects from those of the FDA. The different legal frameworks and political mandates of the two agencies can therefore result in differing regulatory timelines and/or approval decisions [12,13]. We found that 12 of the 29 breast cancer drugs in our analysis did not possess a dual approval for this tumor entity based on both the FDA and EMA review. While six of these drugs had already gained non-centralized European approvals in the pre-EMA era, the other six cases reflect a genuinely different approval status between the United States and Europe. Among these, bevacizumab (with market authorization for breast cancer in Europe, but no equivalent approval in the United States) is the most prominent and most recent example [17]. Importantly, we found no further major divergences in regulatory outcomes for breast cancer drugs during the last ten years covered in our analysis (i.e., from 2009 to 2018; the two drugs approved by FDA in 2018, olaparib and talazoparib, both received an EU approval in the first half of 2019.) Our findings are in agreement with a recent analysis of new drug applications processed by the FDA and EMA between 2014–2016, which found 98% of final decisions to be concordant between the two agencies [13]. The increasing convergence of regulatory outcomes probably reflects a heightened harmonization of drug regulatory processes between the U.S.A. and Europe as well as more globally coordinated development and registration efforts by internationally active pharmaceutical companies.

Nevertheless, the U.S.A. and Europe clearly have distinct approaches to the approval of breast cancer drugs. This is highlighted by the statistically highly significant differences in the application of special regulatory designations and pathways that we found. Since 1995, more than three quarters of the breast cancer drugs approved in the U.S.A. benefited from one or more of these measures, compared to less than 10% of European approvals. The delayed introduction of some of these regulatory programs in Europe (e.g., the FDA’s priority review in 1992 vs. the EMA’s accelerated assessment in 2005) is not by itself sufficient to explain this discrepancy. During the last decade of our analysis (2009–2018), eight of the ten newly approved breast cancer drugs had received special designations from the FDA, but none from EMA. Instead, EMA evidently applies a higher hurdle to award these programs: of all oncology drugs approved in the period from 2007 to 2017, 61% had been granted priority review by the FDA, while only 22% were subject to the corresponding accelerated assessment by the EMA [8]. Likewise, the FDA approved five cancer drugs with breakthrough therapy designation in 2018, compared to only two EMA oncology approvals with the recently introduced European PRIME status that same year [18,19]. Our study shows that the conditional approval of breast cancer drugs, subject to later review of additional clinical data, was a rare event at both the FDA and EMA. Still, the imbalance we observed in our dataset (since 2006, two U.S.A. accelerated approvals versus one EU conditional marketing authorization) conforms to a more general pattern in oncology: the EMA granted only 17 conditional marketing authorizations for oncology products during the first 10 years following the introduction of this pathway in 2006, whereas the FDA gave accelerated approval to 25 new cancer drugs during the same time (own analysis based on the published literature) [20,21]. These findings demonstrate that the EMA has so far been more selective than the FDA in applying expedited development/review designations and pathways, not just to breast cancer drugs, but to new oncology medicines generally. The FDA’s more frequent use of special regulatory pathways has been discussed as a potential risk to high safety and efficacy standards. However, studies on this topic tend to emphasize the overall benefits of this approach [10,21,22].

Across all therapeutic areas, 71% of FDA-approved novel drugs during the period of 2014–2018 were authorized in the United States before any other country (own analysis based on the published literature) [18]. In our cohort, 88.2% of new drugs approved by both the FDA and EMA for breast cancer were first authorized for this use in the U.S.A., including every single breast cancer drug approved after 1997. This finding cannot be explained by a hypothetical advantage of U.S.A. companies in achieving faster FDA approvals; among the 17 dual approvals in our sample, eight breast cancer drugs originated from European pharmaceutical firms, another eight from U.S.A.-based ones, and one from Japan.

One factor explaining this may be the FDA’s broader use of expedited development and review pathways, as described above [23]. In our dataset, a longer European approval process, compared to that in the U.S.A., coincided in all cases but one (docetaxel) with a delayed European approval. This is not self-evident, since the same drug is frequently submitted to the two agencies on different dates, creating separate starting time points for each review process. A recent study of drug approvals across all therapeutic areas found that the FDA review times were on average 60 days shorter than those at the EMA (not even including the additional time span to a positive European Commission decision) [12]. This difference in the overall sample was largely driven by the subset of cancer drugs, for which the median review time even differed by 173 days. Such delayed regulatory decisions have real-life consequences: a recent study calculated that accelerating the approval of a single new cancer drug by one year could lead to thousands of life-years gained for patients. However, the impact for any particular drug will depend on the achievable (overall) survival benefit and the number of eligible patients [24]. Moreover, pricing and reimbursement decisions on the national level frequently cause further delays in patients’ access to newly approved medicines [25,26].

Despite the relatively small number of drugs in our study, we found highly statistically significant differences between the U.S.A. and Europe regarding the use of special regulatory programs, approval dates, and total review times. Since our analysis was limited to first approvals of novel drugs in breast cancer, our findings may, however, not be generalizable to supplementary approvals for additional sub-indications within this tumor entity.

## 4. Materials and Methods

For this analysis, we queried the FDA and EMA regulatory databases to assemble a list of all drugs that had received an approval explicitly mentioning the therapy or prevention of “breast cancer” as an indication between 1 January 1995 and 31 December 2018. We excluded generic and biosimilar drugs as well as combinations of previously approved medicines from our analysis.

For the resulting list of 29 drugs, we extracted the relevant data from initial and subsequent approval letters, prescription information and public assessment reports as well as other publicly available FDA and EMA regulatory documents [27,28], supplemented by information from the published literature and company press releases. The approval dates used in our analysis were those leading to the first (original or supplementary, conditional or standard) marketing authorization in breast cancer, regardless of other previous or subsequent approvals for the same drug in the same geography. For the EU, the date of authorization by the European Commission was used as the approval date. From the review and approval documents and other FDA/EMA databases, reports and publications (e.g., Center for Drug Evaluation and Research, or CDER, list of approved fast track products, annual CDER New Drug Therapy Approvals reports, annual EMA Human Medicines Highlights reports), we determined which of the available expedited development and review programs (five in the U.S.A. and five in the EU) were associated with each drug and which type of approval (conditional or standard) was granted, in as far as the first approval for breast cancer in the respective geography was concerned [27,28]. In the case of accelerated/conditional approvals, the date of the subsequent standard approval, if applicable, was also identified.

For the comparison of the review and approval timelines between the U.S.A. and the EU, we excluded drugs for which the first EU approval predated the establishment of the EMA in 1995 as well as bevacizumab, since it is no longer FDA-approved for use in breast cancer. For the remaining 17 drugs approved following FDA and EMA review, “approval lag” times were calculated as the difference in calendar days between the respective approval dates. Review times were calculated as the number of calendar days between NDA (New Drug Application)/MAA (Marketing Authorization Application) submission and approval. The EMA review times therefore include so-called “clock stops”, during which applicants prepare answers to the questions raised by the agency. Toremifene was excluded from the analysis of review times, since the submission date leading to approval was not available on the FDA server. For U.S.A. approvals, the date on which the FDA received the application (as stated in the approval letter) was used as the submission date. For European approvals, we separately collected the date of the positive CHMP opinion, in order to determine the EMA review time net of the additional time interval to a European Commission decision.

The statistical analysis of results was conducted as follows. Differing frequencies of regulatory pathways were analyzed by Fisher’s exact test and 95% Clopper–Pearson exact confidence intervals were calculated. For review durations and approval times, we analyzed differences using the Wilcoxon test, presenting medians with 95% confidence intervals. *P*-values of less than 0.05 were considered statistically significant. All analyses were performed using IBM SPSS Statistics (Version 26.0.0.0, 2019). Clopper–Pearson confidence intervals were calculated with Cytel Studio (Version 8.0.0, 2007).

## 5. Conclusions

In conclusion, this study showed a high degree of concordance between the FDA and EMA approval decisions for breast cancer drugs, especially since 2009. However, the analysis also revealed a persisting median “approval lag” of about 12 months between the U.S.A. and Europe. In particular, we found that the median time span from regulatory submission to positive FDA/EMA decision was more than twice as long in Europe compared to the U.S.A., even though all post-bevacizumab regulatory decisions were the same or highly similar. 

This work therefore identifies one particular measure the European Union could take in order to accelerate the approval of breast cancer drugs, in keeping with its statement that breast cancer is a “primary focus of EU policy initiatives” [29]. To do so, the EMA could consider applying its existing special regulatory programs (e.g., PRIME status or accelerated assessment) to breast cancer drugs, similar to the FDA’s much broader use of such programs in the United States.

## Figures and Tables

**Table 1:** FDA (Food and Drug Administration) and EMA (European Medicines Agency) new drug approvals for breast cancer (1995–2018).

Generic Name	Brand Name	FDA Approval & Regulatory Designations						EMA Approval & Regulatory Designations				
		Year	Accelerated Approval (AA)	Fast Track (FT)	Breakthrough Therapy (BT)	Priority Review (PR)	Orphan Drug	Year	CMA/ExC	PRIME	Accelerated Assessment	Orphan Drug
**anastrozole**	Arimidex	**1995**	-	-	-	-	-	pre-EMA	n/a	n/a	n/a	n/a
**goserelin**	Zoladex	**1995**	-	-	-	-	-	pre-EMA	n/a	n/a	n/a	n/a
**letrozole**	Femara	**1997**	-	-	-	-	-	pre-EMA	n/a	n/a	n/a	n/a
**epirubicin**	Ellence	**1999**	-	-	-	**PR**	**Orph**	pre-EMA	n/a	n/a	n/a	n/a
**exemestane**	Aromasin	**1999**	-	-	-	-	**Orph**	pre-EMA	n/a	n/a	n/a	n/a
**gemcitabine**	Gemzar	**2004**	-	-	-	**PR**	-	pre-EMA	n/a	n/a	n/a	n/a
**raloxifene**	Evista	**2007**	-	-	-	-	**Orph**	not BC	n/a	n/a	n/a	n/a
**ixabepilone**	Ixempra	**2007**	-	-	-	**PR**	-	none	n/a	n/a	n/a	n/a
**doxorubicin non-PEG**	Myocet	none	n/a	n/a	n/a	n/a	n/a	**2000**	-	-	-	-
**ibandronic acid**	Bondronat	not BC	n/a	n/a	n/a	n/a	n/a	**2003**	-	-	-	-
**doxorubicin PEG**	Doxil	not BC	n/a	n/a	n/a	n/a	n/a	**2003**	-	-	-	-
**docetaxel**	Taxotere	**1996**	**AA(+)**	-	-	PR	-	**1995**	**ExC(+)**	-	-	-
**toremifene**	Fareston	**1997**	-	-	-	-	**Orph**	**1996**	-	-	-	-
**capecitabine**	Xeloda	**1998**	**AA(+)**	-	-	**PR**	-	**2001**	-	-	-	-
**trastuzumab**	Herceptin	**1998**	-	**FT**	-	**PR**	-	**2000**	-	-	-	-
**fulvestrant**	Faslodex	**2002**	-	-	-	-	-	**2004**	-	-	-	-
**paclitaxel (alb. bound)**	Abraxane	**2005**	-	**FT**	-	-	-	**2008**	-	-	-	-
**lapatinib**	Tykerb	**2007**	-	**FT**	-	**PR**	-	**2008**	**CMA(+)**	-	-	-
**bevacizumab**	Avastin	**(2008)**	**AA(−)**	-	-	**(PR)**	-	**2007**	-	-	-	-
**eribulin**	Halaven	**2010**	-	**FT**	-	**PR**	-	**2011**	-	-	-	-
**pertuzumab**	Perjeta	**2012**	-	-	-	**PR**	-	**2013**	-	-	-	-
**everolimus**	Afinitor	**2012**	-	-	-	-	-	**2012**	-	-	-	-
**ado-trastuzumab emt.**	Kadcyla	**2013**	-	**FT**	-	**PR**	-	**2013**	-	-	-	-
**palbociclib**	Ibrance	**2015**	**AA(+)**	-	**BT**	**PR**	-	**2016**	-	-	-	-
**ribociclib**	Kisqali	**2017**	-	-	**BT**	**PR**	-	**2017**	-	-	-	-
**neratinib**	Nerlynx	**2017**	-	-	-	-	-	**2018**	-	-	-	-
**abemaciclib**	Verzenio	**2017**	-	**FT**	**BT**	**PR**	-	**2018**	-	-	-	-
**olaparib**	Lynparza	**2018**	-	-	-	**PR**	-	**2019**	-	-	-	-
**talazoparib**	Talzenna	**2018**	-	-	-	**PR**	-	**2019**	-	-	-	-
**TOTAL**	**29**	**25**	**3**	**6**	**3**	**15**	**4**	**21**	**2**	**0**	**0**	**0**

Orph = orphan drug; CMA = conditional marketing authorization, ExC = marketing authorization under exceptional circumstances; none = no approval; not BC = no approval for breast cancer; pre-EMA = approval pre-dates EMA; (+) = converted to standard approval; (–) = not converted to standard approval; non-PEG = non-PEGylated liposomal; PEG = PEGylated liposomal; alb. bound = albumin bound; emt. = emtansine; bevacizumab not included in total numbers for FDA.
